# Supplementary material for: High Potency of SN-38-Loaded Bovine Serum Albumin Nanoparticles Against Triple-Negative Breast Cancer
Source: Pharmaceutics. 2019 Nov 1;11(11):569. doi: 10.3390/pharmaceutics11110569 (PMC6920977; doi:10.3390/pharmaceutics11110569)
Supplement: Supplementary file 1 [file pharmaceutics-11-00569-s001.pdf]

pharmaceutics

MDPI

Supplementary Materials: High Potency of SN-38-Loaded Bovine Serum Albumin Nanoparticles Against Triple-Negative Breast cancer

Hsin-Che Lin, Chih-Hung Chuang, Meng-Hsuan Cheng, Yu-Chih Lin and Yi-Ping Fang

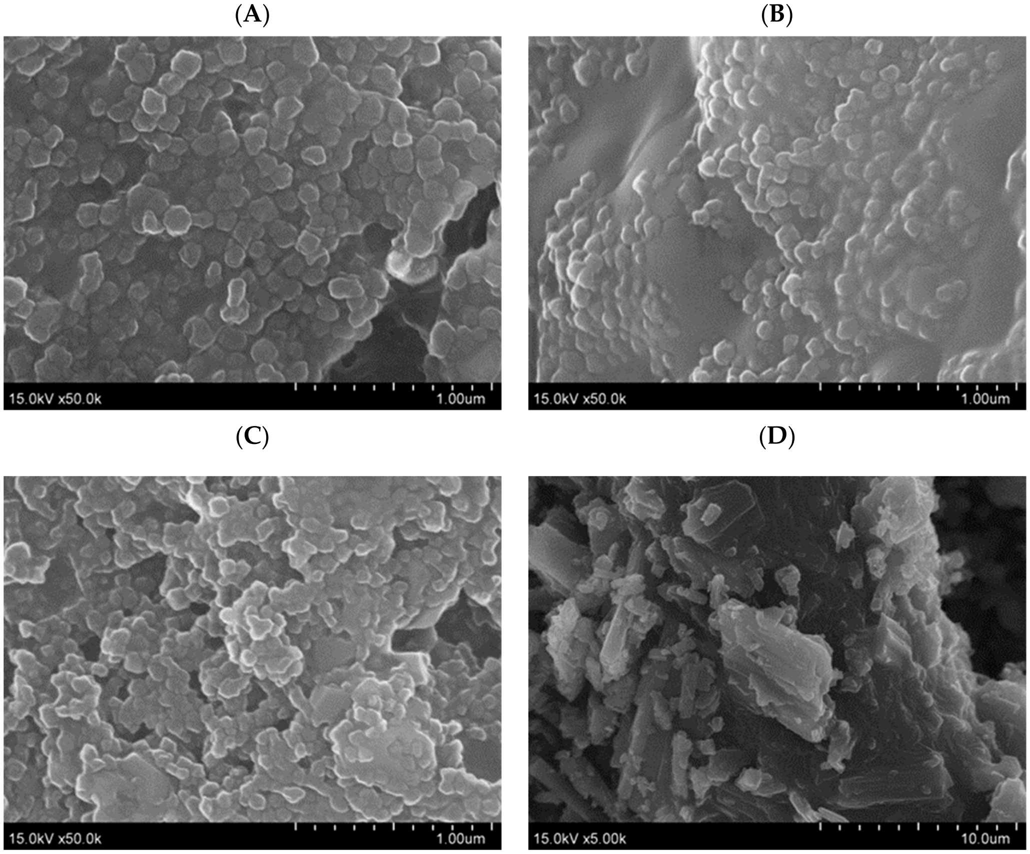

Figure S1. SEM images of the (**A**) sBSANP-F75 (**B**) sBSANP-F62.5 (**C**) sBSANP-F40 (**D**) SN-38 crystals. Note: (**A**)–(**C**) maganification 500,000×; (**D**) maganification 5000×.

Table S1. models and coefficients for the in vitro release of SN38 control group and SN-38-loaded bovine serum albumin nanoparticles (sBSANP) with different albumin concentration in various liposomal formulations.

	Zero Order	Frist Order	Huguchi	Korsmeyer–Peppas	Hixson-Crowell
	$C = C_0 - Kt$	$\log C = \log C_0 - \frac{K \cdot t}{2.303}$	$Q = \sqrt{D(2C - C_s)C_s t}$	$f = \frac{M}{M_\infty} = K \cdot t^n$	$\sqrt[3]{W_0} = \sqrt[3]{W} + K_{HC} t$
Release model	C_0 is the initial concentration of drug. K is first order release constant.	C_0 is the initial concentration of drug K is first order release constant.	Q is the amount drug released per unit area at time t. D is the diffusion coefficient in the matrix. C is the initial amount of drug in the matrix. C_s is the solubility of drug in the matrix.	f is the amount of drug released. M_∞ is the amount of drug at the equilibrium state. M is the amount of drug released over time t. K is rate constant. n is the release exponent related to the drug release mechanism.	W_0 is the initial amount of drug in the system. W is the amount remaining in the system at time t K_{HC} is rate constant for Hixson-Crowell equation.
Control	y = 4.8427x + 1.9323<br>R^2 = 0.9994	y = -0.0317x + 2.0105<br>R^2 = 0.9914	y = 20.141x - 14.599<br>R^2 = 0.9758	y = 0.8666x + 0.8271<br>R^2 = 0.9988 (n = 0.867)	y = 0.0978x - 0.0069<br>R^2 = 0.9974
sBSANP-F75	y = 2.3714x + 2.5079<br>R^2 = 0.9985	y = -0.0124x + 1.9921<br>R^2 = 0.9988	y = 0.234x + 0.8558<br>R^2 = 0.9781	y = 0.7368x + 0.6709<br>R^2 = 0.9972 (n = 0.723)	y = 0.0416x + 0.0325<br>R^2 = 0.9991
sBSANP-F62.5	y = 2.0629x + 2.4022<br>R^2 = 0.9998	y = 2.0629x + 2.4022<br>R^2 = 0.9998	y = 2.0629x + 2.4022<br>R^2 = 0.9998	y = 2.0629x + 2.4022<br>R^2 = 0.9998 (n = 0.668)	y = 2.0629x + 2.4022<br>R^2 = 0.9998
sBSANP-F40	y = 2.2961x + 1.2785<br>R^2 = 0.9993	y = −0.0118x + 1.9975<br>R^2 = 0.9958	y = 0.2607x + 0.6317<br>R^2 = 0.971	y = 0.7696x + 0.5903<br>R^2 = 0.9853 (n = 0.770)	y = 0.0398x + 0.0133<br>R^2 = 0.9974

© 2019 by the authors. Submitted for possible open access publication under the terms and conditions of the Creative Commons Attribution (CC BY) license (http://creativecommons.org/licenses/by/4.0/).
